# Microsatellite Instability in Urine: Breakthrough Method for Bladder Cancer Identification

**DOI:** 10.3390/biomedicines12122726

**Published:** 2024-11-28

**Authors:** Manuel Alejandro Rico-Méndez, María de la Luz Ayala-Madrigal, Anahí González-Mercado, Melva Gutiérrez-Angulo, Jorge Adrián Ramírez de Arellano Sánchez, Saul Armando Beltrán-Ontiveros, Betsabe Contreras-Haro, Itzae Adonai Gutiérrez-Hurtado, José Miguel Moreno-Ortiz

**Affiliations:** 1 Doctorado en Genética Humana, Instituto de Genética Humana “Dr. Enrique Corona Rivera”, Departamento de Biología Molecular y Genómica, Centro Universitario de Ciencias de la Salud, Universidad de Guadalajara, Guadalajara 44340, Jalisco, Mexico; manuel.rico8557@alumnos.udg.mx (M.A.R.-M.); luz.ayala@academicos.udg.mx (M.d.l.L.A.-M.); anahi.gonzalez@academicos.udg.mx (A.G.-M.); 2 Departamento de Ciencias de la Salud, Centro Universitario de los Altos, Universidad de Guadalajara, Tepatitlán de Morelos 47600, Jalisco, Mexico; melva.gutierrez@academicos.udg.mx; 3 Instituto de Investigación en Ciencias Biomédicas, Departamento de Biología Molecular y Genómica, Centro Universitario de Ciencias de la Salud, Universidad de Guadalajara, Guadalajara 44340, Jalisco, Mexico; adrian.ramirez@academicos.udg.mx; 4 Centrode Investigación y Docencia en Ciencias de la Salud, Universidad Autónoma de Sinaloa, Culiacán Rosales 80030, Sinaloa, Mexico; saul.beltran@uas.edu.mx; 5 Unidad de Investigación Biomédica 02, Unidades Médicas de Alta Especialidad, Hospital de Especialidades, Centro Médico Nacional de Occidente, Instituto Mexicano del Seguro Social, Guadalajara 44329, Jalisco, Mexico; betsabecoha@gmail.com; 6 Departamento de Biología Molecular y Genómica, Centro Universitario de Ciencias de la Salud, Universidad de Guadalajara, Guadalajara 44340, Jalisco, Mexico

**Keywords:** bladder cancer, microsatellite instability, exfoliated bladder tumor cells, marker, microsatellite instability analysis, urine, pembrolizumab

## Abstract

Bladder cancer (BC) is the most common neoplasm of the urinary system and ranks tenth in global cancer incidence. Due to its high recurrence rate and the need for continuous monitoring, it is the cancer with the highest cost per patient. Cystoscopy is the traditional method for its detection and surveillance; however, this is an invasive technique, while non-invasive methods, such as cytology, have a limited sensitivity. For this reason, new non-invasive strategies have emerged, analyzing useful markers for BC detection from urine samples. The identification of tumor markers is essential for early cancer detection and treatment. Urine analysis offers a non-invasive method to identify these markers. Microsatellite instability (MSI) has been proposed as a promising marker for tumor cell detection and guided targeted therapies. Therefore, this review aims to explore the evidence supporting the identification of MSI in exfoliated bladder tumor cells (EBTCs) in the urine, emphasizing its potential as a non-invasive and clinically effective alternative for tumor identification. Furthermore, establishing clinical guidelines is crucial for standardizing its application in oncological screening and validating its clinical utility.

## 1. Introduction

Bladder cancer (BC) is the most prevalent neoplasm affecting the urinary system, characterized by a high mutation rate and genomic instability [1]. With a global incidence of 3% and a mortality rate of 2.2%, BC ranks as the tenth most common cancer and the thirteenth leading cause of cancer-related deaths worldwide [2]. In 2020, BC accounted for over half a million cases, with projections indicating that this number could double by 2040 [3].

BC is classified histopathologically into low-grade and high-grade types and based on tumor invasion into non-muscle invasive bladder cancer (NMIBCa) or muscle-invasive bladder cancer (MIBCa). NMIBCa comprises 75% of BC cases and primarily develops in the mucosal layer, exhibiting high recurrence rates. Molecularly, it is identified by deletions in 9q and mutational signatures linked to the APOBEC protein family [4]. In contrast, MIBCa, accounting for 25% of cases, manifests when tumors infiltrate deeper bladder layers, including the muscular and adventitial layers, and has high metastasis rates [5]. Notably, MIBCa has the third-highest mutation rate among all cancers and is characterized by alterations in genes related to DNA damage repair (DDR) pathways [4].

BC is associated with the highest lifetime cost per patient among all cancers, largely due to its high recurrence rate and the need for continuous surveillance [6]. The standard detection and surveillance method involves cystoscopy and cytology. However, these techniques have their limitations [7], leading recent research to identify new urinary biomarkers for BC detection, many of which are Food and Drug Administration (FDA)-approved; however, none have been incorporated into clinical practice guidelines, nor demonstrated clinical utility [6].

Microsatellite instability analysis (MSA) stands out as a promising candidate for inclusion, which is FDA-approved [8] and has been integrated into the clinical practice guidelines for cancers such as colorectal [9], endometrial [10], and gastric cancer [11], but not for BC. The inclusion of this analysis in clinical guidelines is due to the significant benefit that patients with microsatellite instability (MSI)-positive tumors derive from treatment with pembrolizumab. This monoclonal antibody targets and blocks programmed cell death protein 1 (PD-1), an immune checkpoint expressed on T cells, which plays a key role in regulating the immune response. By interrupting the interaction with its ligand, programmed death-ligand 1 (PD-L1), present in tumor cells, the effector capacity of T cells is reactivated, allowing them to recognize and attack tumor cells [12,13]. Therefore, the main goal of this review is to analyze the evidence supporting the detection of MSI in bladder tumor cells present in urine, in order to support the development of clinical guidelines that standardize its use as a genomic alterations test associated with BC and confirm its clinical relevance.

## 2. Traditional Techniques for Bladder Cancer Detection

The primary symptom of BC is hematuria. Therefore, a urologist must first rule out any potential external causes. If these results are negative, an imaging test is then ordered, along with a cystoscopy to examine the bladder mucosa for any bladder masses. If bladder masses are identified, a tissue sample is obtained through biopsy, or alternatively, a transurethral resection of the bladder tumor is performed for histopathological confirmation and staging. A method that accompanies cystoscopy is cytology, which involves the cytomorphological analysis of a urine sample to detect tumor cells [14]. This loss of cell adhesion is indicative of a high degree of malignancy [15].

Despite cystoscopy and cytology being standard techniques for BC detection, they have limitations. Cystoscopy is an invasive method with a high cost that brings complications for the patient [7]. These include anxiety prior to the procedure, pain during and after the intervention, and temporary alterations in sexual function, such as a reduced libido in both sexes and erectile dysfunction in men. However, these effects are typically short-lived and tend to disappear within a few weeks after the procedure [16]. On the other hand, cytology is a non-invasive method with a limited sensitivity for detecting high-grade tumors (64%). In addition, it has a low sensitivity for detecting low-grade tumors (12%) [17]. Furthermore, inflammatory conditions of the bladder, such as infections or prior procedures, can significantly influence cytology results. Additionally, the reproducibility of this technique is limited, and its reliability heavily depends on the expertise of the technician interpreting the findings [18]. Due to the need to improve the study of BC, there is a search for new non-invasive technologies for the detection of useful genetic markers for the diagnosis, progression, and selection of treatments for cancer. One promising candidate is liquid biopsy, which includes blood, saliva, and urine tests. Urine is an easy resource to obtain, in which compounds such as nucleic acids, proteins, leukocytes, lymphocytes, epithelial cells, kidney cells, and urothelial cells are found [19]. Because the cells of bladder tumors can exfoliate, urine can harbor bladder tumor cells, which can be isolated for analysis. In addition, urine has low amounts of protein and high amounts of free circulating tumor DNA and RNA, which can be used as target markers for the diagnosis of BC [20]. Therefore, several panels have been proposed using urine samples as their subject of study (Table 1), and some of them have been approved by the FDA.

The use of urinary markers has been explored as a non-invasive tool for the management of BC. In recent years, various panels of markers have been developed, some of which are FDA-approved, with multiple clinical purposes, including supporting diagnosis, detecting BC, and identifying alterations in nucleic acids and proteins associated with this neoplasm, monitoring the disease, and evaluating recurrence (Table 1).

Although these panels are highly innovative and enhance the accuracy of BC cell detection, none of them have been included in clinical practice guidelines, and their clinical utility has not been fully demonstrated [6]. Taking advantage of these weaknesses is where MSA can come into play.

## 3. Microsatellite Instability Development

Microsatellites, also known as short tandem repeats, are tandem sequences of a repetitive unit with a size of one to six base pairs. They are distributed throughout the human genome, with a higher frequency in intronic regions. They represent 3% of the human genome, and most of them are polymorphic [43,44]. Due to the repetitive nature of microsatellites, DNA polymerases lead to replication slippage, causing insertions or deletions (indels) in their sequences. The system responsible for correcting these mismatch mistakes is the Mismatch Repair System (MMR), which is mainly composed of the proteins MLH1, MSH2, MSH6, MSH3, and PMS2 [45].

**Figure 1:**
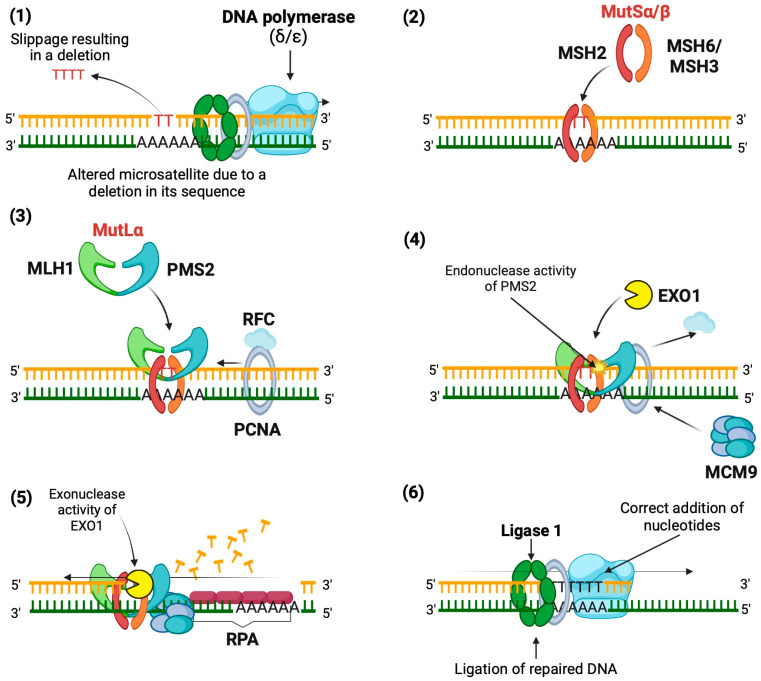
Mechanism of action of the Mismatch Repair System. Mechanism of DNA repair using a microsatellite deletion as an example. (1) A base mismatch occurs due to slippage by DNA polymerase. (2) The mismatch is recognized by MutSα or MutSβ, depending on the size of the indel. (3) MutLα is recruited to form a repair complex with PCNA and RFC. (4) PMS2 is activated to create entry sites for EXO1. (5) EXO1 excises the nascent strand around the mismatch. (6) The gap is resynthesized and sealed by DNA polymerase and DNA ligase 1Created in BioRender.

The repair mechanism, using a deletion in a microsatellite as an example, is carried out according to the following steps (Figure 1).

1. Base mismatch: The DNA polymerase (δ or ε, depending on the DNA strand being polymerized) causes a slippage error in the microsatellite sequence, resulting in a deletion.
2. Mismatch recognition: The MutSα heterodimer (MSH2 and MSH6) or MutSβ heterodimer (MSH2 and MSH3) binds to mismatches in the DNA, depending on the size of the mismatch. MutSα recognizes SNVs and indels of 1 to 2 bp, while MutSβ identifies larger indels. This binding induces a conformational change that allows MutSα/β to move along the DNA.
3. Recruitment of MutLα: MutSα/β facilitates the recruitment of MutLα (MLH1 and PMS2), forming a tetrameric complex along with proliferating cell nuclear antigen (PCNA) and replication factor C (RFC), which binds to the DNA strand.
4. Activation of PMS2: PCNA activates MutLα, enabling PMS2 to exert its endonuclease activity specifically on the nascent strand, creating entry sites for exonuclease 1 (EXO1).
5. DNA excision: EXO1 excises the nascent strand from the mismatch to a gap in the single-stranded DNA, with the help of helicase MCM9, forming a protein complex (MutSα/MutLα/EXO1/MCM9).
6. Resynthesis and ligation: Replication protein A (RPA) protects the single-stranded DNA generated, while polymerase correctly resynthesizes the DNA, and finally, DNA ligase 1 seals the gap [46,47,48,49,50,51].

However, if any of the proteins that compose this system has deficient activity or is not expressed, especially MLH1, the repair process is deficient. This causes the accumulation of indels in microsatellite sequences, altering their allelic size, resulting in a phenotype known as MSI [41].

MSI is classified into the following three categories based on the number of altered loci in the analyzed marker panel: high MSI (MSI-H), when alterations are observed in two or more loci; low MSI (MSI-L), when only one locus shows changes; and microsatellite stability (MSS), when no alterations are detected in any loci [52].

Recent research has demonstrated that patients with MSI-H tumors exhibit a better prognosis in response to immunotherapy compared to those with MSI-L or MSS tumors [53]. This has led to the exploration of specific treatment options for patients with these molecular markers, with pembrolizumab emerging as a leading therapy [8].

A series of trials, collectively known as KEYNOTE studies and sponsored by Merck, the developer of pembrolizumab, have focused on evaluating its efficacy across various cancers characterized by deficiencies in the MMR system (dMMR) and/or MSI-H. Among these, KEYNOTE-016 was one of the first trials to investigate pembrolizumab’s impact on MSI-H solid tumors, particularly in colorectal cancer (CRC) [8]. This was followed by a second phase, known as the KEYNOTE-164 trial, which reported an overall survival rate of 33% in MSI-H CRC patients, demonstrating improved outcomes compared to the prior treatments received by the participants [54]. The series culminated in the phase 3 KEYNOTE-177 trial, which compared pembrolizumab to standard chemotherapy in metastatic MSI-H CRC patients, showing a significant improvement in progression-free survival [55]. These trials were instrumental in highlighting the importance of MSI as a marker for predicting response to immunotherapy, which led, in 2017, to FDA approval for the therapeutic use of this monoclonal antibody for oncology patients, both adults and pediatric oncology patients, with solid tumors exhibiting these genetic markers. This was the first time a treatment was approved based on the presence or absence of a single marker [8], and it is now approved in more than 80 countries as a treatment for various types of cancer, including BC [12].

The efficacy of pembrolizumab has also been tested in non-colorectal MSI-H/dMMR-positive cancers. The phase 2 KEYNOTE-158 trial assessed pembrolizumab in 27 different cancer types, with endometrial, gastric, pancreatic, and cholangiocarcinoma cancers being the most frequently studied. The trial reported high response rates and sustained durations of response among patients who benefited from the treatment, further emphasizing pembrolizumab’s potential as an effective therapeutic alternative for MSI-H/dMMR cancer patients [56].

## 4. Role of Microsatellite Instability in Pembrolizumab Response for MSI-H Tumors

The reason why pembrolizumab is effective in patients with MSI-H tumors lies in its inhibition of immune checkpoints (PD-1/PD-L1). MSI-H tumors have a high mutational burden, which leads to the production of tumor-specific neoantigens [8,57]. This triggers a heightened response from antigen-specific cytotoxic T cells. However, prolonged exposure to these antigens causes T cells to enter a state of exhaustion, reducing their cytotoxic capacity and allowing tumor growth. This negative feedback mechanism is driven by inhibitory receptors on T cells that, upon binding to their ligands, limit the effector phase, their clonal expansion, and function, thereby preventing damage to healthy cells [13,58].

PD-1 is an immune checkpoint receptor primarily expressed on activated T cells, B cells, natural killer cells, macrophages, and dendritic cells. It regulates the immune response by preventing the overactivation of T cells, which could otherwise harm healthy tissues. This function is achieved by binding to its ligands, PD-L1, which is expressed in various cell types, including tumor cells, and PD-L2 (programmed death-ligand 2), expressed on immune cells such as dendritic cells and macrophages [13,58].

When PD-1 binds to PD-L1 or PD-L2, it triggers intracellular signaling by phosphorylating its tyrosine inhibitory domain. This phosphorylation recruits the SHP2 phosphatase, which dephosphorylates key proteins involved in T cell activation, primarily the T cell receptor (TCR). This dephosphorylation reduces the activation and effector function of T cells, inhibiting the immune response against tumor cells. Thus, the interaction between PD-1 and its ligands represents a mechanism by which tumors can evade the immune system, allowing them to grow and spread uncontrollably [13,59]. Tumor cells exploit this pathway by overexpressing PD-L1, inhibiting T cell responses, and preventing them from attacking. This phenomenon is accentuated in MSI-H tumors, where T cells are primed to recognize tumor antigens. However, they become inactivated due to PD-1 signaling [13,58].

**Figure 2:**
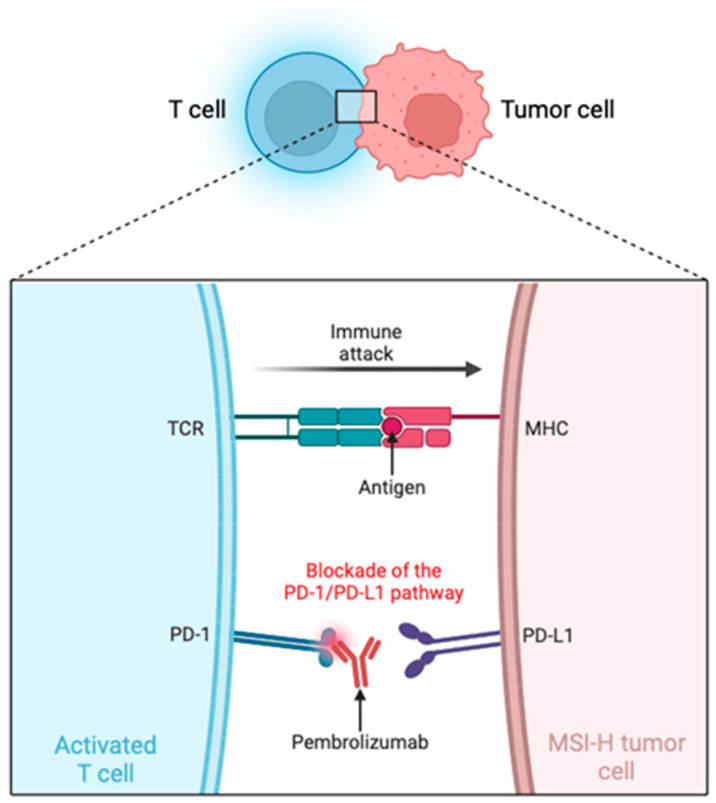
Blockade of PD-1/PD-L1 signaling in tumor immunotherapy. Created in BioRender. TCR: T cell receptor; MHC: Major Histocompatibility Complex; PD-1: Programmed cell death protein 1; PD-L1: Programmed death-ligand 1; MSI-H: High microsatellite instability.

Pembrolizumab, commercially known as “Keytruda”, is a humanized IgG4 Kappa monoclonal antibody targeted against PD-1 on T cells present in tumors, blocking their interaction with PD-L1 on tumor cells. This interrupts the inhibitory signaling pathway and consequently reactivates T cell-mediated immunity, enabling these cells to effectively recognize and attack tumor cells (Figure 2). Therefore, blocking the PD-1/PD-L1 pathway significantly enhances the antitumor response in patients with tumors, promoting tumor regression and improving clinical prognosis. Additionally, the inhibition of the PD-1 pathway can increase cytokine production, further boosting the immune response against tumors [12,13].

Pembrolizumab has demonstrated a significant efficacy in the treatment of patients with MSI-H CRC, both in early stages [60] and advanced stages [55], offering better overall response and higher progression-free survival rates compared to chemotherapy. Additionally, it reduces the number of severe adverse events [55].

## 5. Role of Microsatellite Instability in Bladder Cancer Development

MSI is involved in the onset, progression, and prognosis of various types of cancer [53]. It is predominantly represented in CRC, found in 15% of sporadic cases and 90% of hereditary cases [61]. Additionally, it has been evaluated across eighteen types of cancer and detected in fourteen of them, including BC [62].

The finding of MSI in BC is likely to be due to alterations in the DDR pathways, which is a hallmark of BC, especially of the MIBCa subtype [4], involving repair mechanisms such as double-strand breaks (DSBs), homologous recombination, non-homologous end joining, alternative end joining, base excision repair, nucleotide excision repair (NER), and MMR [63]. The pathways with stronger evidence in bladder carcinogenesis are the NER and DSB pathways [64]. While current evidence on the relationship between the MMR pathway and bladder tumorigenesis remains limited, this area presents an opportunity for further research. Recent studies, including those by Fraune et al. (2020) and Mohamedali et al. (2022), highlight that the loss of expression or reduction in the activity of MMR proteins in BC occurs at a very low frequency, underscoring the potential stability of this pathway in the disease [65,66]. On the other hand, research by Catto et al. (2003), Burger et al. (2006), and Vageli et al. (2013) demonstrates higher frequencies of a reduced expression of MMR proteins in bladder tumors [67,68,69]. Although information about the involvement of the MMR pathway in bladder carcinogenesis is limited, there are additional studies investigating MSI in bladder tumor tissue. These studies report frequencies with a wide range of variation, as shown in Table 2.

## 6. Microsatellite Instability in Urine-Derived Bladder Tumor Cells

**Figure 3:**
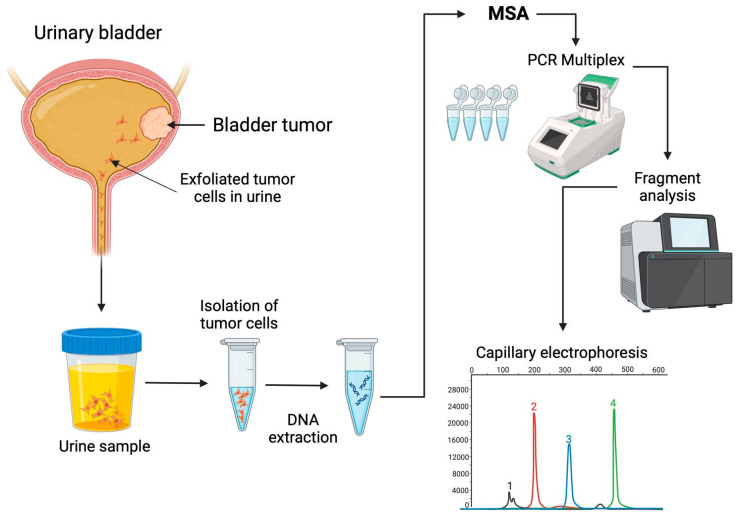
Microsatellite instability analysis in exfoliated bladder tumor cells in urine. In the lower-right corner of the figure is an illustrative image of capillary electrophoresis showing the peaks resulting from the amplification of DNA fragments. The numbered peaks (1, 2, 3, and 4) represent amplified fragments of different sizes detected through capillary analysis. The Y-axis represents relative fluorescence units (RFU), and the X-axis corresponds to base pairs (bp). It should be noted that this figure is purely illustrative and does not represent specific experimental data. Created in BioRender.

Since MSI is a characteristic of tumor cells, and these cells may lose their cell adhesion capacity, it is possible to recover them from the urine sediment of patients with bladder tumors, extract the DNA, and perform MSA (Figure 3) [7,73,74].

The collection, handling, and storage of urine samples are crucial steps in ensuring reliable results for identifying urinary biomarkers, particularly those derived from exfoliated bladder tumor cells (EBTCs) in urine. For optimal collection, samples should come from patients with an active tumor in the bladder. The sample must be placed in a sterile container and should be the first void of the day [73].

As previously noted, urine contains a diverse range of components that can degrade if not processed promptly. Therefore, it is recommended that urine samples be stored at 4 °C and processed within 4 h of collection [73]. To isolate exfoliated urothelial cells, 30 to 50 mL of urine is typically required. The sample should be centrifuged to obtain a pellet, which is then washed with saline solutions. DNA extraction is performed from the cellular pellet following specific protocols, enabling the execution of MSA [73,74,75,76].

For better quality control of assays, it has been recommended to filter urine samples using a membrane filter with 8 μm diameter pores. This filter captures urothelial cells, which are typically larger than 20 μm, and removes smaller cells present in the urine [74].

In some cases, urine preservatives may be employed to maintain the composition and integrity of cells and nucleic acids. This is particularly important because microbial growth may occur between sample collection and processing, and preservatives can prevent this. Additionally, preservatives allow the sample to be stored at room temperature [75]. For the long-term storage of urine samples intended for MSA, they can be stored at −80 °C for up to 3 years [76].

Some research groups have performed MSA on EBTCs in urine, reporting variable MSI frequencies (Table 3).

In addition to reporting MSI frequencies in urine samples, some authors mentioned in Table 3 compared MSA with cytology, demonstrating that MSA is more sensitive for detecting BC compared to cytology [77,78,79,81].

This can be explained by the fact that several patients included in these studies had low-grade tumors and, as previously mentioned, cytology has a low sensitivity for detecting such tumors due to the lower number of tumor cells present in urine [83]. However, this limitation does not apply to MSA, which can detect MSI even when the tumor cell content in the sample is as low as 5–10% [84]. Furthermore, it has been reported that MSA in urine samples has an overall sensitivity ranging from 75% to 96% compared to cytology, whose sensitivity ranges from 13% to 50%. It is important to note that MSA is equally effective in identifying both low- and high-grade tumors [7].

Similarly, comparisons were made between the MSI findings in urine samples and tumor DNA, with concordance reported between the MSI results in both types of samples [72,77]. Moreover, it has been demonstrated that MSA can detect bladder tumor cells in urine sediment and predict recurrence before it is detected by cystoscopy [85].

A wide range of MSI frequencies can be observed in tumor tissue DNA (Table 2), varying from 1.1% to 72.7%, compared to frequencies obtained from urine samples (Table 3), which range from 43.5% and 97%. This disparity may be attributed to the differing sensitivities of the panels used in each study, as each panel is unique. As discussed in the following section, there is a close relationship between the nature of the markers used and their sensitivity for detecting MSI. These variations emphasize the importance of further optimizing MSA in urine samples, which could make them as reliable as tissue samples for MSI detection in BC.

## 7. Discussion and Conclusions

The detection of MSI in EBTCs from urine samples has emerged as a promising non-invasive method for identifying BC. Traditional diagnostic approaches, such as cystoscopy and cytology, while widely used, present significant limitations. Cystoscopy is an invasive and costly procedure, which can cause discomfort for patients, and cytology, though non-invasive, has a limited sensitivity, particularly in detecting low-grade tumors [7]. These limitations have driven the search for new, more accurate assessment methods.

Urinary markers, including MSI, offer the potential to enhance the detection and monitoring of BC by providing a non-invasive, easily accessible diagnostic tool [7]. MSA has already demonstrated its clinical value in other cancers, such as colorectal, endometrial and gastric cancers, where it has been incorporated into clinical practice guidelines [9,10,11]. In BC, MSA could serve not only as a disease marker, but also as a guide for personalized treatment.

To ensure accurate MSI detection, it is crucial to use a highly sensitive marker panel. The panels mentioned in Table 3 do not report their sensitivity. However, the markers analyzed vary according to the type of repeat they contain. It is well known that the sensitivity of a marker, as well as its predictive value, decreases as the number of nucleotides in the repeats increases; in other words, they have a polymorphic nature [86,87]. Therefore, an ideal panel for MSA should include non-polymorphic mononucleotide markers, which would ensure more reliable results in the detection of MSI, especially when the goal is to guide specific treatments for cancer patients.

Although several research teams have used multiple markers to analyze MSI in urine samples from patients with BC, to date, there is no validated marker panel due to technical limitations regarding the selection of optimal alleles and the interpretation of results [7]. However, these limitations represent an opportunity for future research to guide the development of an effective marker panel for BC screening, which would benefit patients through these advancements.

There are precedents that may guide the selection of useful markers. MSA panels have been validated in other types of cancer using tumor tissue DNA. However, their results can serve as a foundation to explore their utility in detecting MSI in urine samples.

In 1997, the National Cancer Institute recommended a panel of five markers, which included three dinucleotide markers (D2S123, D5S346, and D17S250) and two mononucleotide markers (BAT-25 and BAT-26). However, this panel had some limitations. The use of dinucleotide markers required comparing the results of MSA in the DNA from tumor tissue and adjacent tissue from each patient to classify MSI [52]. This can be challenging in some types of cancer, where obtaining healthy tissue is difficult. Years later, a new panel for MSA known as the “pentaplex panel” was introduced, consisting of five quasi-monomorphic mononucleotide markers (NR-21, NR-24, NR-27, BAT-26, and BAT-25), proposed by Suraweera et al. (2002) and Buhard et al. (2006). The term “quasi-monomorphic” refers to the fact that the allele sizes of these markers are nearly identical across most populations worldwide. Unlike polymorphic markers, which can introduce biases in analysis due to the variability in the allele sizes among populations, this variation does not indicate MSI, but rather a natural difference in allele size [87,88].

In 2010, Goel et al. evaluated the pentaplex panel and reported a sensitivity of 95.6% for detecting MSI, with a predictive value of 100%. Furthermore, when using this highly sensitive panel, it is not required to compare MSI results with reference DNA. Instead, a range of quasi-monomorphic variation is used to determine the MSI status of each marker. Despite its advantages, the pentaplex panel has not gained widespread acceptance due to a limited understanding of its technical procedure and the lack of reports supporting its validity in laboratories, as well as the fact that its validation has only been tested in CRC. Additionally, it was reported that the markers NR-24 and BAT-25 had a lower predictive power compared to the other three markers. To address this drawback, a triplex panel was proposed with NR-21, NR-27, and BAT-26, which increased the sensitivity of the analysis to 97.4%. However, this panel was unable to detect cases of CRC with deficiencies in MSH6 [52].

To address these limitations, the BAT-52, BAT-59, and BAT-62 markers were added to the pentaplex panel according to Chung et al. (2023), which resulted in an increased sensitivity for detecting MSI in colorectal, endometrial, and gastric cancers [89]. Additionally, Kang et al. (2021) proposed a triplex panel with the CAT-25, BAT-40, and BAT-26 markers, with the advantage that CAT-25 can detect cases of CRC with MSH6 deficiencies, unlike the pentaplex panel. To validate these markers, they used 300 samples from 60 types of cancer, including urothelial cancers such as BC [90]. Therefore, this triplex panel could be a promising option for the detection of MSI in BC.

However, despite the promising data on MSA in urine samples, it is necessary to investigate the identification of highly sensitive and specific markers for effective MSI detection in urine samples, with the aim of establishing a panel for BC screening and, with the necessary validations, integrating it into clinical practice for the management of this neoplasm.

Previously, some KEYNOTE trials were highlighted that validated the efficacy of pembrolizumab across various cancer types. For BC, the phase 2 KEYNOTE-057 clinical trial evaluated the efficacy of pembrolizumab in patients with high-risk NMIBC who were unresponsive to Bacillus Calmette–Guérin (BCG) therapy, as the PD-1 pathway plays a role in BCG resistance. Out of 334 patients screened, 96 met the inclusion criteria and demonstrated a favorable response in terms of antitumor activity. However, 13 patients developed severe treatment-related adverse effects, including arthralgia and hyponatremia [91]. It is important to note that this trial did not include patients with MSI-H, highlighting the need for further investigation of the MSI pathway in BC. Such studies would help validate the use of pembrolizumab not only in tumor tissue DNA, but also in EBTCs in urine, potentially opening up new avenues for more targeted detection, monitoring, and treatment strategies for these patients.

Given this gap in research, there is a clear need for additional large-scale studies to validate the clinical utility of MSI in this context, especially because the use of immunotherapies such as pembrolizumab can bring adverse effects to patients, including impacts in the cutaneous, gastrointestinal, endocrine, neurological, and respiratory systems, among other alterations [13]. Therefore, it is important to select candidate patients based on these genetic markers, as the risks associated with its administration may not justify the benefits in the absence of a significant clinical response.

Such studies would help to determine its effectiveness as a reliable and non-invasive diagnostic tool, as well as its potential role in guiding therapeutic decisions. If successfully validated, the incorporation of MSA as a urine marker in BC detection could improve early diagnosis, reduce the need for invasive procedures, and lead the way for more personalized therapeutic approaches, ultimately improving patient outcomes.

## Figures and Tables

**Table 1:** Urinary markers for BC management.

Panel	Principle	Detection Unit	Sensitivity	Specificity	References
Exfoliated bladder tumor cells					
UroVysion *	FISH	Aneuploidies in chromosomes 3, 7, and 17 and deletions in 9p21	73%	83%	[21,22]
Immunocyt *	F-LMab	Carcinoembryonic antigen and sulfated mucin glycoproteins	60–100%	75–84%	[23]
URO17	IHC	Cytokeratin 17	100%	92.6%	[24]
Cell detect	Immunostaining	Malignant cells	82.8%	88.2%	[25]
Proteins					
BTA-STAT *	ICA	Proteins associated with human complement factor H	76.7%	67.9%	[26,27]
BTA TRAK *	ELISA	Proteins associated with human complement factor H	58%	73%	[21,28]
NMP22 *	ELISA	NMP22 protein	50–70%	60–80%	[21,29]
UBC Rapid test	ELISA	Cytokeratins 8 and 18	72.2%	79.4%	[24,30]
ADXBLADDER	ELISA	MCM5 protein	75.6%	71.1%	[31]
BLCA-4	ELISA	BLCA-4 protein	93%	97%	[32]
Oncuria^TM^	Multiplex Immunoassay	A1AT, APOE, ANG, CA9, IL8, MMP9, MMP10, PAI1, SDC1, and VEGFA proteins	93%	93%	[33]
mRNA					
Xpert Bladder Cancer Monitor	RT-PCR	mRNA levels of *CRH*, *IGF2*, *UPK1B*, *ANXA10*, and *ABL1* genes.	75%	77%	[34]
CxBladder	RT-PCR	mRNA levels of *MDK*, *HOXA13*, *CDC2*, *IGFBP5*, and *CXCR2* genes	100%	75%	[35]
DNA alterations					
UroMuTERT	Sequencing	Mutations in *TERT* promoter	87.1%	94.7%	[36]
Uromonitor-V2^®^	qPCR	Mutations in *TERT*, *FGFR3*, and *KRAS* genes	93.1%	85.4%	[37]
uCAPP-Seq	Sequencing	DNA alterations in regions from 460 genes	83%	97%	[38]
DNA methylation					
P3 panel	qMSP	Methylation status of *PCDH17*, *POU4F2*, and *PENK* genes	84%	100%	[39]
Bladder Care	qMSP	Methylation status of TRNA-Cys, *SIM2* and *NKX1-1*	93.5%	92.6%	[40]
EpiCheck	qMSP	Methylation status of 15 loci	68.2%	88%	[41]
UroMark	Targeted bisulfite sequencing	Methylation status of 150 loci	96%	97%	[42]

FISH: Fluorescence In Situ Hybridization; F-LMab: Fluorescence-labeled monoclonal antibodies; IHC: Immunohistochemistry: BTA: Bladder tumor antigen; ICA: Immunochromatography; ELISA: Enzyme-Linked Immunosorbent Assay; RT-PCR: Reverse Transcription Polymerase Chain Reaction; qMSP: Quantitative Methylation-Specific Polymerase Chain Reaction; *: FDA approval.

**Table 2:** Reports of MSI frequencies in tumor tissue of patients with BC.

n	MSI Frequency	Analyzed Markers	Reference
44	72.7%	BAT-26, BAT-40, BAX, TGFβ, IGFIIR, MSH3, D2S123, D9S283, D9S1851, and D18S58	[70]
448	1.1%	BAT-25, BAT-26, D2S123, D5S346, D17S250, and MYCL1	[65]
220	39%	ACTBP2, D16S310, D16S476, D18S51, D4S243, D9S162, D9S171, D9S747, FGA, INF-a, MBP, and MJD	[71]
70	65.7%	GSN and D18S51	[72]
84	8%	BAT25, BAT26, D17S250, D2S123, D5S346, BAT40, D10S197, MYC1L, D18S58, D18S69, TGFβR2, BAX, hMSH3, and hMSH6	[67]
117	8.5%	BAT25, BAT26, D2S123, APC, and D17S250	[68]

**Table 3:** Reports of MSI frequencies in urine samples of patients with BC.

n	MSI Frequency	Analyzed Markers	Reference
20	95%	ACTBP2, D16S310, FGA, D21S1245, D4S243, D16S476, D9S747, D18S51, MBP, MJD, D9S162, IFNA, and D9S171	[77]
12	83%	D4S243, FGA, ACTBP2, D9S162, D9S171, D9S747, IFNA, MJD52, D16S310, D16S476, D18S51, MBP, and D21S1245	[78]
34	97%	D4S243, FGA, ACTBP2, D8S307, IFNA, D9S162, D9S171, D9S747, D11S488, THO, D13S802, MJD, D16S310, D16S476, D17S695, D17S654, D18S51, MBP, D20S48, and D21S1245	[79]
47	94%	D4S243, FGA, ACTBP2, D9S162, D9S171, D9S747, IFNA, MJD52, D16S310, D16S476, D18S51, MBP, and D21S1245	[80]
150	74%	D9S747, D9S171, D9S162, IFNA, and D4S243	[81]
50	76%	D9S63, D9S156, and D9S283	[82]
220	43.5%	ACTBP2, D16S310, D16S476, D18S51, D4S243, D9S162, D9S171, D9S747, FGA, INF-a, MBP, and MJD	[71]
